# Exploring the Use of Pegylated Liposomal Doxorubicin (Caelyx^®^) as Pressurized Intraperitoneal Aerosol Chemotherapy

**DOI:** 10.3389/fphar.2019.00669

**Published:** 2019-06-25

**Authors:** Manuela Robella, Marco Vaira, Monica Argenziano, Rita Spagnolo, Roberta Cavalli, Alice Borsano, Sergio Gentilli, Michele De Simone

**Affiliations:** ^1^Unit of Surgical Oncology, Candiolo Cancer Institute, IRCCS—FPO, Candiolo, Italy; ^2^Department of Drug Science and Technology, University of Torino, Torino, Italy; ^3^General Surgery Unit, Department of Health Science, University of Eastern Piedmont, Novara, Italy

**Keywords:** peritoneal carcinomatosis, locoregional chemotherapy, PIPAC, pegylated liposomes, doxorubicin

## Abstract

**Background:** Peritoneal carcinomatosis is a common metastatic pattern in ovarian, gastric, colorectal, and appendiceal cancer; systemic chemotherapy is the current standard of care for peritoneal metastatic disease; however, in a subset of patients its beneficial effect remains questionable. More effective perioperative chemotherapy is needed.

**Materials and methods:** Pressurized intraperitoneal aerosol chemotherapy (PIPAC) is a new treatment that applies chemotherapeutic drugs into the peritoneal cavity as an aerosol under pressure. It’s a safe and feasible approach that improves local bioavailability of chemotherapeutic drugs as compared with conventional intraperitoneal chemotherapy. Till now the drugs used in PIPAC for the treatment of the peritoneal carcinomatosis (PC) are cisplatin, doxorubicin, and oxaliplatin; as of yet, there are no *in vivo* data comparing different drug formulations and dosage schedules of PIPAC. Pegylated liposomal doxorubicin 1.5 mg/sm was aerosolized in PIPAC procedures.

**Results:** Pharmacokinetics analysis of 10 procedures performed with conventional doxorubicin solution at the dose of 1.5 mg/m^2^ were compared to 15 procedures with the same dose of pegylated liposomal doxorubicin (PLD).

Significant differences between experimental groups were detected by one-way ANOVA followed by Bonferroni correction; a p value < 0.05 was considered statistically significant. A statistically different doxorubicin tissue concentration was observed for the doxorubicin solution compared to pegylated liposomal doxorubicin in the right parietal peritoneum and right diaphragm. In the Caelyx^®^ series a mean tissue concentration of 1.27 ± 1.33 mg/g was reported, while in the second one we registered a mean concentration of 3.1 ± 3.7 mg/g.

**Conclusions:** The delivery of nano-particles in PIPAC was feasible, but pegylated liposomal concentrations are lower than standard doxorubicin formulation. Probably mechanical and physical properties of pressurized aerosol chemotherapy might alter their stability and cause structural disintegration.

## Introduction

Peritoneal carcinomatosis (PC) is a clinical presentation both of different primary tumors (synchronous or metachronous) such as colon-rectum, ovarian, appendix, stomach, pancreas, liver, and primitive peritoneal neoplasms such as diffuse peritoneal mesothelioma and primary peritoneal adenocarcinomas. Historically, PC was considered an end-stage pathology and treated by palliative intent with debulking surgery and/or systemic chemotherapy, usually with poor results. However, in the last decades, the treatment of this peculiar cancer spread recorded a growth both in interest and technical improvements, drawing new outlines in the management of PC. Improvement in the treatment of PC follows the two main carcinomatosis features; indeed, some PC are amenable to combined treatment (systemic and loco-regional) with curative intent (or long-term survival expectations) and some others are, still nowadays, treated by palliative approach. Curative approach is, unfortunately, reserved to a minority of selected patients, while most of patients are still treated with palliative intent (due to comorbidity, advanced stage of disease, age limits, availability of specialized centers). The edge between those two major PC features is sometimes unclear and new ways to treat the “gray-zone patients,” merging low-impact treatment and acceptable clinical results may be, probably, the near-future ideal approach to PC.

As reported in literature, intraperitoneal chemotherapy (IPC) seems to be effective (Ceelen and Flessner, 2010), but pharmacological limitation such as poor drug distribution inside the abdominal cavity (bulky disease and adhesions) and technical problems related to the intraperitoneal catheter are responsible for an high rate of patients’ inability to complete the expected IPC cycles (Dedrick and Flessner, 1997; Wright et al., 2015; Yamaguchi et al., 2015). One potential way to avoid the pharmacokinetic limitations of IPC is to apply IPC as a pressurized aerosol, taking advantage of the physical properties of gas and pressure (Alkhamesi et al., 2005) to spray a drug solution. On this base, PIPAC (pressurized intraperitoneal aerosol chemotherapy) has been recently introduced as a new approach to perform IPC; this treatment is based on the observation that under-pressure application of chemotherapy enhances tumor drug uptake (Jacquet et al., 1996; Esquis et al., 2006). PIPAC may be a way to increase distribution and penetration depth of IPC and achieved a superior distribution on the peritoneum and a better penetration into peritoneal nodules compared to conventional IPC in *ex vivo* models (Solass et al., 2012). The size of the particles has a major influence on their behavioral properties and the aerosol particle radius or diameter is a key property used to characterize aerosol. The micropump generates aerosol droplets with a mean diameter of 11 μm; droplet size is heterogeneous ranging from 3 to 15 μm after sedimentation.

So far, the drugs used in PIPAC are cisplatin, doxorubicin, and oxaliplatin but there are no data comparing different drugs and dosage schedules of PIPAC in patients with PC.

Nanoparticles with their sub-micron size, versatility of physical, chemical properties, and easily modifiable surface are uniquely poised to bypass the body clearing systems. They penetrate through extra-and intracellular barriers to deliver drugs into cancer cells thereby enhancing chemotherapeutic efficacy. Specific manipulation of the chemical composition of nanoparticles can significantly increase peritoneal residence time, thereby prolonging exposure of tumor to chemotherapeutic agents will increase the local drug concentration is primary goal of IPC (González-Moreno et al., 2010).

Pegylated liposomal doxorubicin (Doxil^®^ or European trademark Caelyx^®^) is a formulation of doxorubicin in poly(ethylene glycol)-coated (stealth) liposomes with a prolonged circulation time and unique toxicity profile. Doxil^®^ liposomes retain the drug payload during circulation and accumulate preferentially in tissues with increased microvascular permeability, as often is the case of tumors. The potential of Doxil^®^ in the treatment of numerous cancer types and the opportunities it offers in combination with other drugs and therapeutic modalities are under active investigation.

Studies of nanoparticle–mucus interactions reveal that mucus interferes with penetration of nanoparticle through hydrogen bonding, adhesion, and electrostatic interactions (Lai et al., 2009). PEG coating has been used to minimize mucus–nanoparticle interaction thereby increasing nanoparticle penetration through mucus (Maisel et al., 2015).

The promising enhancement of the effectiveness of anticancer drugs encapsulate in nanoparticulate systems in a considerable aspect. Moreover, increased residence time, prolonged drug release over time, and decrease of adverse side effect are notable features of PIPAC application that could increase the positive features of Caelyx® (Nowacki et al., 2017). PIPAC therapeutic outcomes could be remarkably improved exploiting the combination with nanoparticulate systems.

Anyway, there have been only a few published papers that have fully referred to the potential use and initial experimental effects of nanoparticles and Near Infrared Irradiation (NIR) for Hyperthermic IntraPEritoneal Chemotherapy (HIPEC) and none for PIPAC. One of the first publications addressing this topic is detailed review that suggested that the NIR/nanoparticle concept could improve HIPEC (Wu et al., 2015). To our knowledge, only one paper refers to the use HIPEC with pegylated liposomal doxorubicin (PLD) following maximal cytoreduction in patients with advanced abdominal-only gastrointestinal or gynecologic malignancies without any major postoperative complications (Harrison et al., 2008).

The aim of this study is to use doxorubicin in liposomal form in PIPAC procedure in order to assess feasibility, safety, and the possibility of greater penetration.

## Material and Methods

### Material

PLD (Caelyx^®^) is a formulation of doxorubicin in polyethylene glycol (PEG) coated Stealth^®^ liposomes (Gabizon, 2001). “Pegylation” is the process whereby the doxorubicin-containing liposomes are enclosed within a PEG layer; pegylation protects the liposomes from detection by the mononuclear phagocyte system and provides a stabilization effect that reduces adhesion to cells, blood vessel walls, and other surfaces. During circulation, at least 90% of PLD remains encapsulated within the liposomes, resulting in an extended half-life compared to conventional doxorubicin (Alberts et al., 2004). The active ingredient of the formulation is doxorubicin.

Doxorubicine hydrochloride solution was adriblastina 50 mg/1 Vial by Pfizer.

### Methods

PIPAC procedure was performed under general anesthesia and capnoperitoneum (12 mm Hg, 37°C) using a single-port *access* placed in the midline. Explorative laparoscopy allowed peritoneal cancer index (PCI) evaluation; parietal biopsies and ascites removal were performed. The increased intra-abdominal pressure due to the capnoperitoneum increases the drug penetration into tumoral tissues. Using a micropump (Capnopen) the pressurized drug-containing solution was delivered into the abdominal cavity; doxorubicin or liposomal doxorubicin (1.5 mg/sm in 50 ml 0.9% NaCl). The aerosol was kept in a steady-state for 30 min and then exhausted through a closed filter system. At the end of the procedure tissue samples of the peritoneum were recovered from at least three abdominal quadrants.

Between June 2015 and December 2018, 206 PIPAC applications in 104 patients were performed.

For this study we considered 15 PIPAC procedures using Caelyx^®^ at the dose of 1.5 mg/sm. Patients of this subgroup were treated within the framework of an off-label program. Access to this off-label study was limited to patients who had a life-threatening disease with no satisfactory alternative therapies or could not enter a clinical trial. Data were compared with those obtained in 10 patients subjected to PIPAC with the same standard doxorubicin dosage. This sample of patients was taken as a control group from a larger patients’ cohort treated within the ongoing PI-CaP Protocol (number NCT02604784; EudraCT number 2015-000866-72).

All procedures were performed according to the principles of the Helsinki declaration; all patients were extensively informed and sign a consent inform.

Peritoneal samples were collected after 30 min of PIPAC treatment and frozen. Subsequently the tissue samples were homogenized and extracted and the drug concentration was determined by a High Performance Liquid Chromatography (HPLC) analysis.

### Determination of Doxorubicin Tissue Concentration

Tissue extracts were prepared by adding in a test tube one volume of methanol and two volumes of 1 M Tris buffer with pH 8.5 to the tissue. The mixtures were homogenized using polytron (Kinematica GMBH, Eschbach, Germany), and the tissue homogenates were kept on ice for 15 min before adding seven volumes of acetonitrile. The mixtures were vortexed and allowed to stand at room temperature for 15 min before removing the precipitated proteins by centrifugation at 3,000 × *g* for 5 min. After centrifugation, clear supernatants were assessed for doxorubicin content by HPLC using the method described below.

### HPLC Quantitative Determination of Doxorubicin

Quantitative determination of doxorubicin was carried out by an HPLC system consisting of a pump (Shimadzu LC-9A PUMP C) equipped with fluorescence detector (Chrompack). Analyses were performed using an Agilent TC C18 column (250 mm × 4.6 mm, 5 µm). The mobile phase was a mixture of KH_2_PO_4_ 0.01 M (pH 1.4), acetonitrile, and methanol (65:25:10 v/v/v), degassed and pumped through the column with a flow rate of 1 ml/min. The column effluent was monitored at excitation and emission wavelengths of 480 and 560 nm, respectively. The external standard method was used to calculate the drug concentration. For this purpose, 1 mg of doxorubicin was weighed, placed in a volumetric flask, and dissolved in water to obtain a stock standard solution. This solution was then diluted using the mobile phase, providing a series of calibration solutions, subsequently injected into the HPLC system. Linear calibration curve was obtained over the concentration range of 5–100 ng/ml with a regression coefficient of 0.999.

### Determination of Doxorubicin Plasma Concentration and Side Effects

No traces have been found in the peripheral blood for both doxorubicin and Caelyx^®^.

Postoperative mortality was nil. No differences were found between the two treatment groups. No patient reported major complications; in four cases (16%) minor complications were registered (nausea, abdominal pain). There were no alterations in hepatic and renal function, even after repeated PIPAC procedures. The median hospital stay was 2.3 days.

### Statistical Analysis

Data are expressed as the mean ± standard error of the mean (SEM). Significant differences between experimental groups were detected by one-way ANOVA followed by Bonferroni correction using GraphPad InStat software (San Diego, CA, USA). A p value < 0.05 was considered statistically significant.

## Results

This study aimed at the *in vivo* evaluation the tissue distribution of liposomal doxorubicin (Caelyx^®^) in abdominal cavity after PIPAC treatment in comparison to that of doxorubicin solution. Previous researches reported the distribution pattern and penetration depth of doxorubicin solution after PIPAC in postmortem swine model (Khosrawipour et al., 2016; Khosrawipour et al., 2017; Bellendorf et al., 2018).

Here, 24 tissue patient samples using Caelyx^®^ and 39 using doxorubicin solution were collected and analyzed by HPLC analysis. Interestingly the drug penetrates and accumulates in the abdominal cavity tissues, without reaching the systemic circulation in detectable amount. These pharmacokinetic results confirm the loco-regional treatment and represent an advantage for decreasing adverse effects.

**Figure 1:**
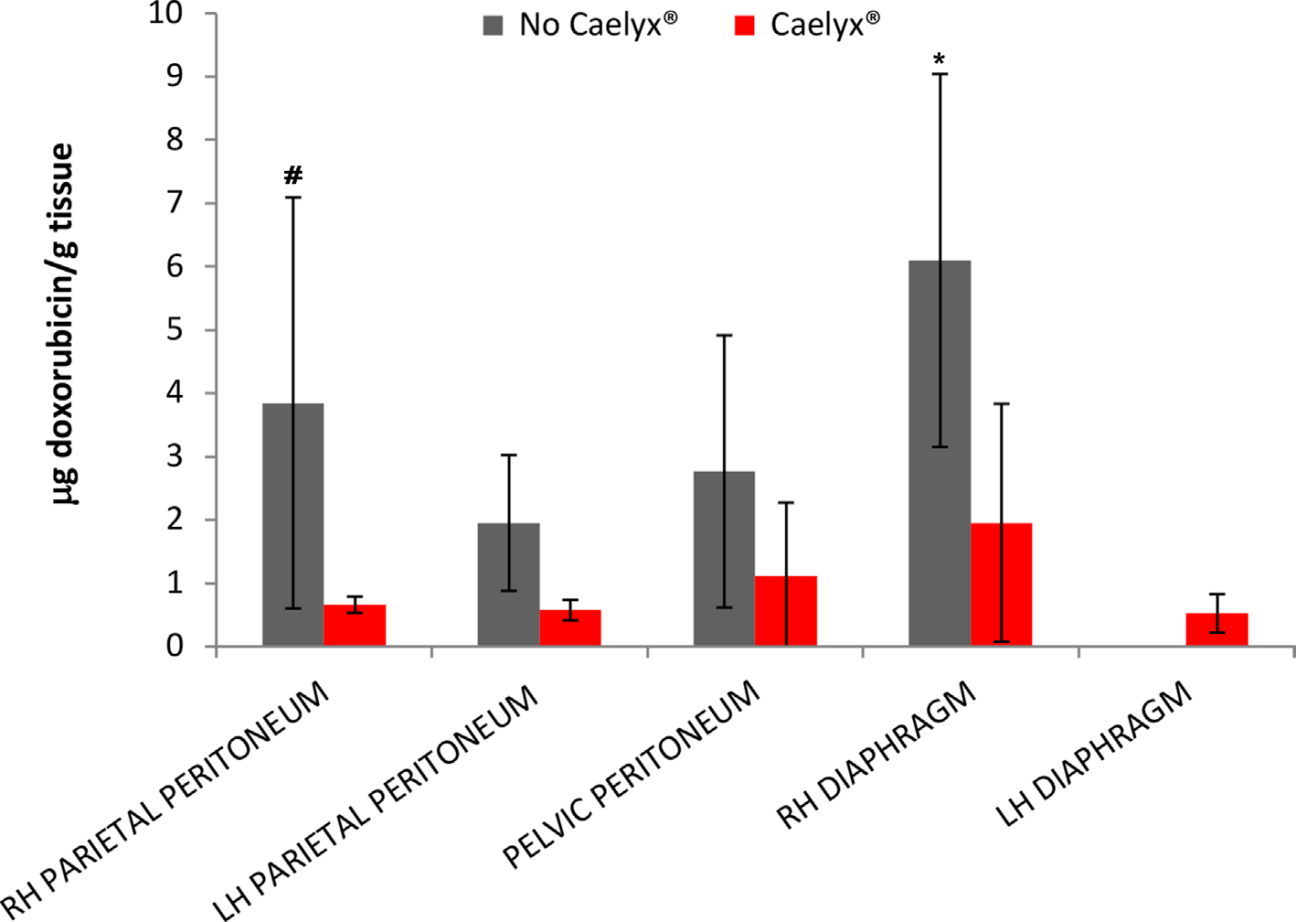
Amount of doxorubicin and pegylated liposomal doxorubicin in the different tissues (µg drug/g tissue). Results are expressed as mean ± SD. ^#^p < 0.05 vs. pegylated liposomal doxorubicin, *p < 0.01 vs. pegylated liposomal doxorubicin, by two-way ANOVA followed by Bonferroni post tests.

**Figure 1** showed the concentration of doxorubicin detected in the different tissue samples.

These results indicate a good drug biodistribution in all the tissues and it is in line with the data obtained by Khosarwipour in the *ex vivo* model (Khosrawipour et al., 2016). The maximum of the drug penetration was observed in the area in front of the microinjection pump (MIP) (that for the position of the operators they are usually the right diaphragmatic and parietal peritoneum, except for cases in which the MIP is directed towards the pelvis due to the presence of adhesions).

A statistically different doxorubicin tissue concentration was observed for the doxorubicin solution compared to PLD in the right parietal peritoneum and diaphragm. Overall, a mean tissue concentration of about 1.2 mg/g was observed after the administration of PLD, while it increased (2.5 fold) in the case of doxorubicin solution.

Indeed, the Caelyx^®^ series a mean tissue concentration of 1.27 ± 1.33 mg/g was reported, while in the second one we registered a mean concentration of 3.1 ± 3.7 mg/g. Therapeutic doxorubicin concentrations were observed following the administration of lower doses that generally ranged between 10 and 60 mg/m^2^. The lower tissue accumulation obtained with the PLD compared to the doxorubicin solution is in agreement with the *ex vivo* study of Mikolajczyk et al. (2018). They found minimal or no penetration with liposomal doxorubicin after PIPAC assuming that liposoma coating might inhibit the interaction between drug and peritoneal surface.

This behavior might be also related to the aerosol pressure that can favor the coalescence of the liposomal systems modifying their surface characteristics and drug release profile (Chattopadhyay et al., 2012).

## Discussion

The treatment of PC has evolved extraordinarily over the years from palliative approach to cytoreductive surgery techniques associated or not to different intraperitoneal drug delivery (HIPEC/EPIC). PIPAC is one of the most recent intraperitoneal delivery systems to improve peritoneal drug penetration. Currently PIPAC is still a palliative treatment, but the results obtained in terms of outcome and safety make it a very promising procedure.

The rational for trying nanoparticles in PIPAC is to take advantage of the antitumoral therapy as potential target for longer effectiveness in the peritoneal cavity: in fact, the instillation of a chemotherapy-containing nanoparticle may improve drug retention and the large molecular size of a nanoparticle would suggest slow clearance from the peritoneal cavity (De Smet et al., 2013).

The purpose of our pharmacokinetic study was to measure the penetration of doxorubicin and PLD within the peritoneal tissues after pressurized intraperitoneal aerosol administration. Our results demonstrate that PLD doesn’t penetrate the peritoneum and its release is probably inhibited by liposomal coating. Similar results were also obtained using PLD in HIPEC procedure (Sugarbaker and Stuart, 2019): the retention of PLD within the peritoneal tissues over a 90-min HIPEC is reported to be only approximately 20% and 180 min of HIPEC 40%. The median area under the curve ratio of peritoneal fluid concentration times time as compared to plasma concentration times was over 1,000 and increased with dose escalation from 50 to 100 mg/sm. When PLD was used for EPIC, the area under the curve ratios were similar to the HIPEC one but retention of drug within the peritoneal tissues was maintained for 24 h with approximately 80% drug utilization. The authors concluded that nanoparticle PLD is slowly absorbed into the intraperitoneal tissues and not appropriate for HIPEC, while EPIC is the preferred methodology for administration. Unfortunately, we could not verify the same effect in PIPAC *in vivo* since abdominal drains were not placed and it was not possible to take serial tissue and/or peritoneal fluid samples.

## Conclusions

The preliminary results of this work demonstrated the feasibility of the delivery of PLD as pressurized aerosol (PIPAC). Tissue concentrations are lower than standard doxorubicin formulation. Further studies might optimize drug biodistribution tuning distance and pressure of the aerosol in PIPAC. The reduced doses and the local administration might decrease side effects and improve the quality of life of patients.

## Data Availability Statement

The datasets generated for this study are available on request to the corresponding author.

## Author Contributions

The authors confirm that the final manuscript has been read, and each author’s contribution has been approved by the appropriate author. This paper has been seen and approved by all authors.

## Conflict of Interest Statement

The authors declare that the research was conducted in the absence of any commercial or financial relationships that could be construed as a potential conflict of interest.
